# 2-Nitro­benzyl 2-chloro­acetate

**DOI:** 10.1107/S1600536809031444

**Published:** 2009-09-05

**Authors:** Kai Zhu, Hui Liu, Yan-Hua Wang, Ping-Fang Han, Ping Wei

**Affiliations:** a College of Biotechnology and Pharmaceutical Engineering, Nanjing University of Technolgy, Xinmofan Road No. 5 Nanjing, Nanjing 210009, People’s Republic of China

## Abstract

In the mol­ecule of the title compound, C_9_H_8_ClNO_4_, an intra­molecular C—H⋯O inter­action results in the formation of a near-planar (r.m.s. deviation 0.002 Å) five-membered ring, which is oriented at a dihedral angle of 4.07 (4)° with respect to the adjacent aromatic ring. In the crystal structure, inter­molecular C—H⋯O inter­actions link the mol­ecules into a two-dimensional network.

## Related literature

For a related structure, see: Pyun *et al.* (2001 ▶). For bond-length data, see: Allen *et al.* (1987 ▶).
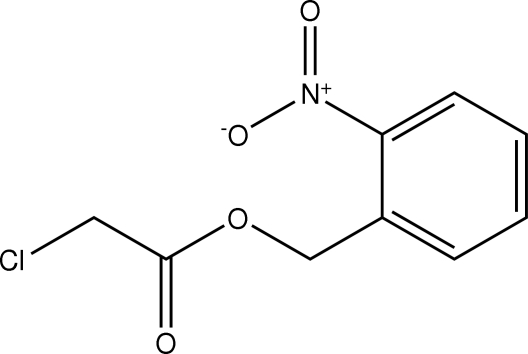


## Experimental

### 

#### Crystal data

C_9_H_8_ClNO_4_
*M* *_r_* = 229.61
Monoclinic, 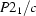

*a* = 8.0270 (16) Å
*b* = 6.7530 (14) Å
*c* = 19.266 (4) Å
β = 92.52 (3)°
*V* = 1043.3 (4) Å^3^
*Z* = 4
Mo *K*α radiation
μ = 0.36 mm^−1^
*T* = 294 K
0.30 × 0.20 × 0.10 mm

#### Data collection

Enraf–Nonius CAD-4 diffractometer
Absorption correction: ψ scan (North *et al.*, 1968 ▶) *T*_min_ = 0.900, *T*_max_ = 0.965
2036 measured reflections
1893 independent reflections
891 reflections with *I* > 2σ(*I*)
*R*_int_ = 0.025
3 standard reflections frequency: 120 min intensity decay: 1%

#### Refinement

*R*[*F* ^2^ > 2σ(*F* ^2^)] = 0.067
*wR*(*F* ^2^) = 0.195
*S* = 1.00
1893 reflections
137 parameters
H-atom parameters constrained
Δρ_max_ = 0.29 e Å^−3^
Δρ_min_ = −0.23 e Å^−3^

### 

Data collection: *CAD-4 Software* (Enraf–Nonius, 1989 ▶); cell refinement: *CAD-4 Software*; data reduction: *XCAD4* (Harms & Wocadlo, 1995 ▶); program(s) used to solve structure: *SHELXS97* (Sheldrick, 2008 ▶); program(s) used to refine structure: *SHELXL97* (Sheldrick, 2008 ▶); molecular graphics: *ORTEP-3 for Windows* (Farrugia, 1997 ▶) and *PLATON* (Spek, 2009 ▶); software used to prepare material for publication: *SHELXL97*.

## Supplementary Material

Crystal structure: contains datablocks global, I. DOI: 10.1107/S1600536809031444/hk2752sup1.cif

Structure factors: contains datablocks I. DOI: 10.1107/S1600536809031444/hk2752Isup2.hkl

Additional supplementary materials:  crystallographic information; 3D view; checkCIF report

## Figures and Tables

**Table 1:** Hydrogen-bond geometry (Å, °)

*D*—H⋯*A*	*D*—H	H⋯*A*	*D*⋯*A*	*D*—H⋯*A*
C1—H1*A*⋯O3^i^	0.97	2.43	3.372 (6)	166
C7—H7*A*⋯O1^ii^	0.93	2.58	3.374 (6)	143

Symmetry codes: (i) 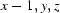; (ii) 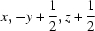.

**Fig. 1.**
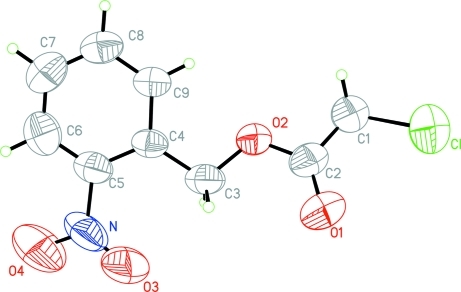
The molecular structure of the title molecule, with the atom-numbering scheme. Displacement ellipsoids are drawn at 30% probability level.

**Fig. 2.**
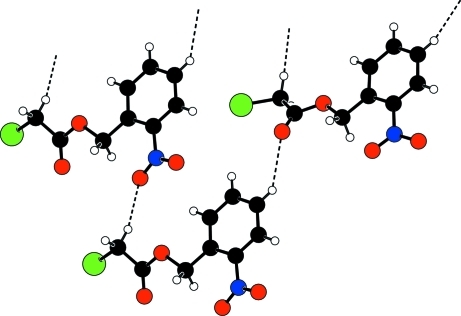
A partial packing diagram of the title compound. Hydrogen bonds are shown as dashed lines.

## supplementary crystallographic information

### Comment

Some derivatives of *p*-nitrobenzyl alcohol are important chemical materials. We report herein the crystal structure of the title compound.

In the molecule of the title compound, (Fig. 1), the bond lengths (Allen *et al.*,  1987) and angles are within normal ranges. Ring A (C4-C9) is, of course, planar. Intramolecular C-H···O interaction (Table 1) results in the formation of a planar five-membered ring B (O2/C3/C4/C9/H9A), which is oriented with respect to the adjacent ring A at a dihedral angle of A/B = 4.07 (4)°.

In the crystal structure, intermolecular C-H···O interactions (Table 1) link the molecules into a two dimensional network (Fig. 2), in which they may be effective in the stabilization of the structure.

### Experimental

For the preparation of the title compound, chloroacetyl chloride (1.1 g) and 2-nitrobenzyl alcohol (1.53 g) were added into the mixture of pyridine (15 ml) and dichloromethane (30 ml) at 273–278 K. The gross products were extracted with n-hexane, washed with water and dried under vacuum, and then recrystallized from dichloromethane. Finally the title compound was obtained (yield; 0.61 g) (Pyun *et al.*, 2001). Crystals suitable for X-ray analysis were obtained by slow evaporation of a methanol solution.

### Refinement

H atoms were positioned geometrically, with C-H = 0.93 and 0.97 Å for aromatic and methylene H, respectively, and constrained to ride on their parent atoms, with U_iso_(H) = 1.2U_eq_(C).

### Crystal data

**Table:**

C_9_H_8_ClNO_4_	*F*(000) = 472
*M**_r_* = 229.61	*D*_x_ = 1.462 Mg m^−^^3^
Monoclinic, *P*2_1_/*c*	Mo *K*α radiation, λ = 0.71073 Å
Hall symbol: -P 2ybc	Cell parameters from 25 reflections
*a* = 8.0270 (16) Å	θ = 9–13°
*b* = 6.7530 (14) Å	µ = 0.36 mm^−^^1^
*c* = 19.266 (4) Å	*T* = 294 K
β = 92.52 (3)°	Block, yellow
*V* = 1043.3 (4) Å^3^	0.30 × 0.20 × 0.10 mm
*Z* = 4	

### Data collection

**Table:**

Enraf–Nonius CAD-4 diffractometer	891 reflections with *I* > 2σ(*I*)
Radiation source: fine-focus sealed tube	*R*_int_ = 0.025
graphite	θ_max_ = 25.3°, θ_min_ = 2.1°
ω/2θ scans	*h* = 0→9
Absorption correction: ψ scan (North *et al.*, 1968)	*k* = 0→8
*T*_min_ = 0.900, *T*_max_ = 0.965	*l* = −23→23
2036 measured reflections	3 standard reflections every 120 min
1893 independent reflections	intensity decay: 1%

### Refinement

**Table:**

Refinement on *F*^2^	Secondary atom site location: difference Fourier map
Least-squares matrix: full	Hydrogen site location: inferred from neighbouring sites
*R*[*F*^2^ > 2σ(*F*^2^)] = 0.067	H-atom parameters constrained
*wR*(*F*^2^) = 0.195	*w* = 1/[σ^2^(*F*_o_^2^) + (0.07*P*)^2^ + 0.84*P*] where *P* = (*F*_o_^2^ + 2*F*_c_^2^)/3
*S* = 1.00	(Δ/σ)_max_ < 0.001
1893 reflections	Δρ_max_ = 0.28 e Å^−^^3^
137 parameters	Δρ_min_ = −0.23 e Å^−^^3^
0 restraints	Extinction correction: *SHELXL97* (Sheldrick, 2008), Fc^*^=kFc[1+0.001xFc^2^λ^3^/sin(2θ)]^-1/4^
Primary atom site location: structure-invariant direct methods	Extinction coefficient: 0.031 (4)

### Special details

**Table:**

Geometry. All e.s.d.'s (except the e.s.d. in the dihedral angle between two l.s. planes) are estimated using the full covariance matrix. The cell e.s.d.'s are taken into account individually in the estimation of e.s.d.'s in distances, angles and torsion angles; correlations between e.s.d.'s in cell parameters are only used when they are defined by crystal symmetry. An approximate (isotropic) treatment of cell e.s.d.'s is used for estimating e.s.d.'s involving l.s. planes.
Refinement. Refinement of *F*^2^ against ALL reflections. The weighted *R*-factor *wR* and goodness of fit *S* are based on *F*^2^, conventional *R*-factors *R* are based on *F*, with *F* set to zero for negative *F*^2^. The threshold expression of *F*^2^ > σ(*F*^2^) is used only for calculating *R*-factors(gt) *etc*. and is not relevant to the choice of reflections for refinement. *R*-factors based on *F*^2^ are statistically about twice as large as those based on *F*, and *R*- factors based on ALL data will be even larger.

### Fractional atomic coordinates and isotropic or equivalent isotropic displacement parameters (Å^2^)

**Table:**

	*x*	*y*	*z*	*U*_iso_*/*U*_eq_	
Cl	−0.3781 (2)	0.3294 (4)	0.19943 (9)	0.1458 (10)	
O1	−0.0286 (4)	0.3868 (6)	0.25207 (18)	0.0985 (12)	
O2	−0.0679 (3)	0.2382 (5)	0.35365 (15)	0.0743 (9)	
O3	0.4281 (5)	0.3113 (9)	0.3977 (2)	0.151 (2)	
O4	0.5546 (5)	0.2492 (8)	0.4935 (3)	0.161 (2)	
N	0.4264 (5)	0.2744 (8)	0.4576 (3)	0.1028 (16)	
C1	−0.2908 (6)	0.2341 (10)	0.2755 (2)	0.1002 (18)	
H1A	−0.3577	0.2745	0.3137	0.120*	
H1B	−0.2939	0.0906	0.2730	0.120*	
C2	−0.1141 (6)	0.2988 (7)	0.2904 (2)	0.0722 (13)	
C3	0.1048 (5)	0.2696 (7)	0.3759 (2)	0.0722 (13)	
H3A	0.1424	0.3976	0.3600	0.087*	
H3B	0.1746	0.1680	0.3566	0.087*	
C4	0.1165 (5)	0.2610 (6)	0.4535 (2)	0.0559 (10)	
C5	0.2673 (5)	0.2629 (7)	0.4927 (2)	0.0706 (13)	
C6	0.2753 (7)	0.2548 (8)	0.5642 (3)	0.0889 (15)	
H6A	0.3780	0.2567	0.5885	0.107*	
C7	0.1300 (8)	0.2440 (7)	0.5994 (3)	0.0874 (15)	
H7A	0.1332	0.2379	0.6477	0.105*	
C8	−0.0187 (6)	0.2423 (7)	0.5624 (2)	0.0735 (13)	
H8A	−0.1173	0.2364	0.5859	0.088*	
C9	−0.0259 (5)	0.2491 (6)	0.4913 (2)	0.0599 (11)	
H9A	−0.1294	0.2456	0.4677	0.072*	

### Atomic displacement parameters (Å^2^)

**Table:**

	*U*^11^	*U*^22^	*U*^33^	*U*^12^	*U*^13^	*U*^23^
Cl	0.1014 (12)	0.224 (2)	0.1105 (13)	0.0086 (13)	−0.0150 (9)	0.0395 (14)
O1	0.101 (3)	0.116 (3)	0.080 (2)	−0.014 (2)	0.0205 (19)	0.024 (2)
O2	0.0632 (18)	0.096 (2)	0.0641 (19)	−0.0135 (17)	0.0092 (14)	0.0080 (17)
O3	0.074 (2)	0.267 (7)	0.115 (3)	−0.035 (3)	0.041 (2)	−0.036 (4)
O4	0.055 (2)	0.222 (6)	0.205 (5)	0.002 (3)	0.001 (3)	0.003 (4)
N	0.049 (3)	0.122 (4)	0.138 (4)	−0.010 (3)	0.013 (3)	−0.032 (4)
C1	0.077 (3)	0.148 (5)	0.076 (3)	−0.006 (4)	0.004 (2)	0.008 (3)
C2	0.080 (3)	0.075 (3)	0.064 (3)	0.004 (3)	0.018 (2)	0.000 (3)
C3	0.062 (3)	0.080 (3)	0.076 (3)	−0.008 (2)	0.021 (2)	−0.005 (3)
C4	0.051 (2)	0.050 (2)	0.068 (2)	0.000 (2)	0.0161 (19)	−0.005 (2)
C5	0.057 (2)	0.071 (3)	0.086 (3)	−0.005 (2)	0.016 (2)	−0.007 (3)
C6	0.081 (3)	0.091 (4)	0.093 (4)	0.003 (3)	−0.015 (3)	0.004 (3)
C7	0.114 (4)	0.080 (4)	0.069 (3)	−0.001 (4)	0.015 (3)	0.001 (3)
C8	0.078 (3)	0.064 (3)	0.080 (3)	−0.003 (3)	0.028 (3)	0.001 (3)
C9	0.056 (2)	0.056 (3)	0.070 (3)	−0.001 (2)	0.0164 (19)	−0.001 (2)

### Geometric parameters (Å, °)

**Table:**

Cl—C1	1.721 (5)	C3—H3B	0.9700
O1—C2	1.189 (5)	C4—C9	1.385 (5)
O2—C2	1.324 (5)	C4—C5	1.398 (6)
O2—C3	1.448 (5)	C5—C6	1.378 (6)
N—O3	1.181 (6)	C6—C7	1.377 (7)
N—O4	1.227 (6)	C6—H6A	0.9300
N—C5	1.472 (6)	C7—C8	1.363 (7)
C1—C2	1.499 (7)	C7—H7A	0.9300
C1—H1A	0.9700	C8—C9	1.369 (6)
C1—H1B	0.9700	C8—H8A	0.9300
C3—C4	1.494 (6)	C9—H9A	0.9300
C3—H3A	0.9700		
			
C2—O2—C3	117.0 (3)	C9—C4—C5	115.6 (4)
O3—N—O4	122.3 (5)	C9—C4—C3	120.8 (4)
O3—N—C5	120.5 (5)	C5—C4—C3	123.7 (4)
O4—N—C5	117.2 (6)	C6—C5—C4	122.7 (4)
C2—C1—Cl	113.6 (4)	C6—C5—N	117.2 (5)
C2—C1—H1A	108.8	C4—C5—N	120.0 (4)
Cl—C1—H1A	108.8	C7—C6—C5	119.5 (5)
C2—C1—H1B	108.8	C7—C6—H6A	120.3
Cl—C1—H1B	108.8	C5—C6—H6A	120.3
H1A—C1—H1B	107.7	C8—C7—C6	119.0 (5)
O1—C2—O2	125.4 (5)	C8—C7—H7A	120.5
O1—C2—C1	126.5 (5)	C6—C7—H7A	120.5
O2—C2—C1	108.1 (4)	C7—C8—C9	121.4 (4)
O2—C3—C4	107.9 (3)	C7—C8—H8A	119.3
O2—C3—H3A	110.1	C9—C8—H8A	119.3
C4—C3—H3A	110.1	C8—C9—C4	121.9 (4)
O2—C3—H3B	110.1	C8—C9—H9A	119.0
C4—C3—H3B	110.1	C4—C9—H9A	119.0
H3A—C3—H3B	108.4		
			
C3—O2—C2—O1	−4.6 (7)	O3—N—C5—C6	−168.6 (6)
C3—O2—C2—C1	174.4 (4)	O4—N—C5—C6	9.6 (8)
Cl—C1—C2—O1	−9.0 (8)	O3—N—C5—C4	11.2 (8)
Cl—C1—C2—O2	172.1 (3)	O4—N—C5—C4	−170.6 (5)
C2—O2—C3—C4	159.9 (4)	C4—C5—C6—C7	0.2 (8)
O2—C3—C4—C9	−7.0 (6)	N—C5—C6—C7	180.0 (5)
O2—C3—C4—C5	172.5 (4)	C5—C6—C7—C8	−0.3 (8)
C9—C4—C5—C6	−0.4 (7)	C6—C7—C8—C9	0.7 (8)
C3—C4—C5—C6	−180.0 (5)	C7—C8—C9—C4	−1.0 (7)
C9—C4—C5—N	179.8 (4)	C5—C4—C9—C8	0.8 (6)
C3—C4—C5—N	0.2 (7)	C3—C4—C9—C8	−179.6 (4)

### Hydrogen-bond geometry (Å, °)

**Table:**

*D*—H···*A*	*D*—H	H···*A*	*D*···*A*	*D*—H···*A*
C1—H1A···O3^i^	0.97	2.43	3.372 (6)	166
C7—H7A···O1^ii^	0.93	2.58	3.374 (6)	143
C9—H9A···O2	0.93	2.27	2.660 (5)	104

Symmetry codes: (i) *x*−1, *y*, *z*; (ii) *x*, −*y*+1/2, *z*+1/2.
